# Case-based review: conservative management of appendicitis – are we delaying the inevitable?

**DOI:** 10.1308/003588412X13171221590296

**Published:** 2012-05

**Authors:** S Davies, A Peckham-Cooper, A Sverrisdottir

**Affiliations:** Burton Hospitals NHS Foundation TrustUK

**Keywords:** Conservative management, Appendicitis, General surgery

## Abstract

Acute appendicitis is a common surgical presentation for which surgical intervention, an appendicectomy, has remained a largely unchallenged primary treatment modality. Traditionally, it has been felt that the pathophysiological progressive nature of appendicitis ultimately leads to perforation. A number of recent studies, however, suggest that the process of appendiceal inflammation may follow a more remitting nature with evidence indicating spontaneous resolution. It is hypothesised that the treatment of uncomplicated appendicitis may therefore be amenable to conservative management with antibiotics. This article aims to highlight some of the issues and challenges relating to the conservative management of acute appendicitis and further demonstrates potential diagnostic and treatment difficulties involved in managing the more unfamiliar condition of recurrent appendicitis.

## Case history

In 2008 a 65-year-old man was admitted via the accident and emergency department to the general surgery team with a short history of right iliac fossa pain. He gave a good history of appendicitis with central abdominal pain localising to the right iliac fossa, some nausea, anorexia and a feeling of being generally unwell. His observations remained within normal limits throughout his admission and within 24 hours of conservative management, analgesia and antibiotics his pain had settled completely.

Ultrasonography was performed during his admission and reported a blind-ended tube in the right iliac fossa, with a diameter of 10mm and appearances consistent with a slightly distended and oedematous appendix. In addition, superior and slightly medial to this was a poorly defined irregular echogenic region measuring up to 50mm across. This was thought to be merely bowel but the possibility of a fluid collection could not be excluded. Given the obvious improvement in his clinical symptoms, he was discharged home with oral antibiotics and an appendicectomy was not performed.

The patient was seen in the outpatient clinic fours weeks after discharge. He remained well, there was no further evidence of a collection or appendix mass on examination and he was discharged from clinic.

The patient re-presented six months later with further generalised abdominal pain, again localising to his right iliac fossa. Routine blood tests were all within normal limits and, once more, his pain settled conservatively over 48 hours and he was discharged home. As a result of his ongoing symp toms, a gastroenterology opinion was sought to exclude the possibility of irritable bowel disease. Computed tomography colonography was reporting as normal with typical appearances of the terminal ileum, caecum and ileocaecal valve. No comment was offered with respect to the appendix and no obvious cause for his pain was identified. He was discharged back to the care of his general practitioner.

In 2011 the patient presented for a third time. He described the same symptoms as previously and furthermore reported that he had been suffering from recurrent, self-limiting episodes on a two-monthly basis for the last three years. There had been no red flag symptoms during this time and he had remained well in the interim period. He had sought medical attention on this occasion due to his pain being more severe, increasingly persistent and unresponsive to analgesia.

Repeat ultrasonography demonstrated an ill defined, fluid filled area in the right iliac fossa, measuring 54mm x 19mm x 22mm. Adjacent to this was a poorly defined, thick walled tubular structure, 52mm long and 18mm wide. A differential diagnosis was proffered suggestive of a collection relating to an inflamed section of bowel, possibly the appendix. On this occasion, the patient deteriorated clinically with high temperatures, tachycardia and localised peritonism. He was taken to theatre and an emergency open appendicectomy performed. The surgical findings included a perforated gangrenous appendix in a pus filled abscess. He made an uneventful recovery and was discharged two days later. Histological examination confirmed the presence of an acute appendicitis and, in addition, reported evidence of an underlying carcinoid in the tip of the appendix with complete resection margins.

## Discussion

In 2010–20H acute appendicitis accounted for over 57,000 acute hospital admissions in England.^1^ The majority of cases are described as uncomplicated but, in approximately 20% of cases, acute appendicitis is ‘complicated’, leading to local or diffuse peritonitis.^2^ Since the first appendicectomy was performed over 120 years ago, it remains the gold standard for the management of uncomplicated appendicitis.

The diagnosis of appendicitis is primarily a clinical one based on a consistent history and clinical presentation. However, radiology has become increasingly used to support the diagnosis. Ultrasonography is considered the first-line radiological investigation for the assessment of complicated appendicitis in which there is a question of an appendix mass or abscess. It is quick, cheap, non-invasive and does not expose the patient to a radiation load. Computed tomography (CT), however, is more sensitive and specific than ultrasonography when diagnosing acute appendicitis and related complications (ultrasonography sensitivity of 86% and specificity of 81% compared with CT sensitivity of 94% and specificity of 95%).^3^

Traditionally, appendicitis is recognised as an acute progressive disease. The cause is usually an obstruction of the lumen of the appendix by a faecalith, lymphoid hyperplasia (such as in inflammatory bowel disease), parasites or, more rarely, a foreign body or tumour.

A faecalith is the most common of these aetiologies, accounting for simple appendicitis in 40%, gangrenous nonperforated appendicitis in 65% and perforated appendicitis in 90% of cases.^4^ The result is a closed system obstruction with an ever increasing interluminal pressure rise as the appendiceal mucosa secretes fluid against the fixed obstruction. Within the closed system, intestinal bacterial load in the appendix multiply, leading to the recruitment of white blood cells, the formation of pus and a subsequent further intraluminal pressure increase. If the obstruction persists, intraluminal pressure rises ultimately above that of the appendiceal veins and leads to venous outflow obstruction. As a consequence, appendiceal wall ischaemia begins, resulting in a loss of epithelial integrity and bacterial invasion of the appendiceal wall.^5^

While initially a localised condition, ongoing pathology may lead to thrombosis of the appendicular artery and veins, ultimately resulting in a perforation, gangrene and necrosis of the appendix.^6^ However, some reports suggest not all cases of appendicitis will progress to the point of perforation and many cases of acute appendicitis will resolve spontaneously.^7^

For over 120 years, since McBurney defined the surgical appendectomy in 1894, it has remained the gold standard for the management of appendicitis. In recent years there has been increasing debate on the methodology of this surgical management, with the advent and development of laparoscopic surgery resulting in the ‘laparoscopic ap pendicectomy’ becoming more widespread. The evidence regarding the choice of surgical intervention remains divided as a number of studies report a reduction in overall length of hospital stay, post-operative pain and morbidity with the laparoscopic approach^8^^–^^9^ but more recent, large-scale randomised controlled trials (RCTs) describing little or no benefit in either management option and benefits being dependent on individual surgical experience.^10^^–^^11^ Whichever surgical management option is used, an emergency appendicectomy is well tolerated by most patients although documented risks of post-operative complications remains variable at between 2% and 25% of patients.^12^^–^^13^

Over the last few years there has been an increasing interest in the idea of conservative management for acute appendicitis. Conservative non-operative antibiotic management of other intra-abdominal inflammations such as uncomplicated diverticulitis is a well established alternative to operative management. Recently, there have been a number of studies including several large randomised trials comparing conservative management of uncomplicated appendicitis with surgery.^14^^–^^18^ Data suggest the use of antibiotic treatment is an effective alternative management option and in fact the authors propose that non-operative management should be first-line.

In 2008 Styrud *et al* performed a multicentre RCT on 252 men presenting with acute appendicitis.^19^ Patients were randomised either to a surgical (laparoscopic or open) or to an antibiotic therapy arm (intravenous antibiotics for two days followed by oral antibiotics for ten days). Of those patients randomised to the antibiotic arm, 86% improved without surgery. The authors concluded that conservative management was a safe alternative for uncomplicated acute appendicitis. However, the rate of recurrence of symptoms of appendicitis among the HI patients treated with antibiotics was 14% during a one-year follow-up period. Furthermore, the diagnosis of acute uncomplicated appendicitis was made following a clinical assessment only and resulted in 14% of patients crossing over to the surgical arm due to complications in the first 24 hours of management.

Similarly, a French study in 2011 randomised 245 patients diagnosed with uncomplicated appendicitis on CT to an antibiotic alone (amoxicillin and clavulanic acid) and a surgical arm.^20^ The authors found little difference in overall outcome data but almost 25% of patients who had initially had symptom resolution following antibiotic therapy had experienced a recurrence of symptoms within 12 months compared with 14% in previous studies.^16^^–^^19^

A meta-analysis in 2010 of the five RCTs comparing surgical and conservative management of acute appendicitis also concluded that although antibiotics may be used as a primary treatment alternative for selected patients with suspected uncomplicated appendicitis, it was unlikely to supersede appendectomy based on the currently available evidence.^21^ Selection bias and patient crossover to surgical arms in the RCTs was cited as the major concern and the authors suggested that the surgical appendicectomy should remain the gold standard therapy for the management of acute appendicitis. A Cochrane systematic review performed in 2011 also found the results inconclusive.^22^

The quality of the studies was low to moderate, a subgroup analysis could not be made and therefore it was also recommended that appendicectomy remains the standard treatment for acute appendicitis.

Despite best available evidence, there continue to be certain situations where, anecdotally, conservative management may play a primary role. In those patients who are considered ‘high risk’ with significant co-morbidities and anaesthetic risk, in those at the extremes of age and in those where surgical intervention is simply unavailable (submariners), conservative management may well be considered the treatment modality of choice. Similarly, patients who are diagnosed with appendicitis early in the disease process (as our patient was) may respond favourably to initial antibiotic therapy.

The evidence, however, remains very limited and is largely case-based in nature. In these cases, the incidence of recurrence following the conservative management of appendicitis must remain a concern. Cases of recurrent appendicitis are well documented in the literature and involve repeated admissions and associated patient morbidity. Studies clearly outline a significant potential for recurrence in those patients treated conservatively, even within an initial limited follow-up period of *12* months. The case presented in this report demonstrates symptomatic recurrence over the extended period of three years and highlights the significant potential for the longer term impact of not performing definitive operative management. Indeed, there are no similar data reporting recurrence rates in conservatively managed patients after *12* months and future studies must incorporate a longer follow-up period.

## Conclusions

There remains a limited body of evidence for the conservative management of appendicitis. While the risk of recurrence is well documented, the authors believe that there may remain a potential role for the use of conservative management in selected patient groups, using antibiotic therapy as a bridge prior to performing definitive elective surgery. In some cases this may be to allow for a period of optimisation, reducing the risks of immediate operative management or, alternatively, it may be used in those patients where surgical intervention is unavailable. Patient selection remains crucial and it is not our opinion that this should be the treatment option of choice for all patients. Further research needs to incorporate long-term follow-up before definitive best practice guidelines can be determined. The question still remains that by managing patients conservatively are we simply just delaying the inevitable?
